# Progestogens Are Metabolized by the Gut Microbiota: Implications for Colonic Drug Delivery

**DOI:** 10.3390/pharmaceutics12080760

**Published:** 2020-08-12

**Authors:** Zoe Coombes, Vipul Yadav, Laura E. McCoubrey, Cristina Freire, Abdul W. Basit, R. Steven Conlan, Deyarina Gonzalez

**Affiliations:** 1 Institute of Life Science 2, Swansea University Medical School, Swansea University, Singleton, Swansea SA28PP, UK; r.s.conlan@swansea.ac.uk; 2 Department of Pharmaceutics, UCL School of Pharmacy, University College London, WC1N 1AX, UK; vipul.yadav@intractpharma.com (V.Y.); laura.mccoubrey.18@ucl.ac.uk (L.E.M.); Cristina.Freire@aptuit.com (C.F.); 3 Kuecept Limited, Potters Bar, Hertfordshire EN6 1TL, UK

**Keywords:** progesterone, medroxyprogesterone, levonorgestrel, large intestine metabolism, colonic stability, steroids, gastrointestinal bacteria, microbiome

## Abstract

Following oral administration, the bioavailability of progestogens is very low and highly variable, in part due to metabolism by cytochrome P450 enzymes found in the mucosa of the small intestine. Conversely, the mucosa in the colon contains much lower levels of cytochrome P450 enzymes, thus, colonic delivery of progestogens may be beneficial. Microbiota in the colon are known to metabolize a great number of drugs, therefore, it is important to understand the stability of these hormones in the presence of colonic flora before developing formulations. The aim of this study was to investigate the stability of three progestogens: progesterone, and its two synthetic analogues, medroxyprogesterone acetate (MPA) and levonorgestrel (LNG), in the presence of human colonic microbiota. Progesterone, MPA, and LNG were incubated in mixed fecal inoculum (simulated human colonic fluid) under anerobic conditions. Progesterone was completely degraded after 2 h, whereas levels of MPA and LNG were still detectable after 24 h. The half-lives of progesterone, MPA, and LNG in fecal inoculum were 28, 644, and 240 min, respectively. This study describes the kinetics of colonic microbial metabolism of these hormones for the first time. MPA and LNG show promise for delivery to the colon, potentially improving pharmacokinetics over current oral delivery methods.

## 1. Introduction

**Figure 1:**
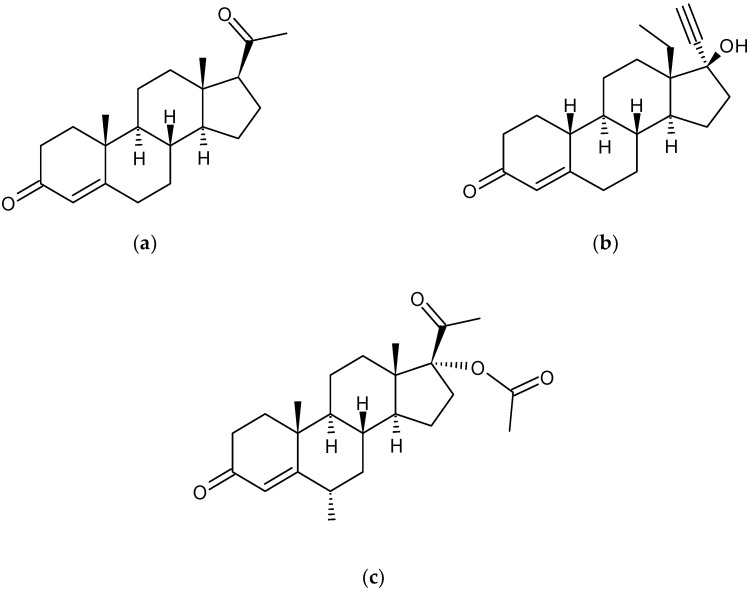
Chemical structures of (**a**) progesterone, (**b**) levonorgestrel, and (**c**) medroxyprogesterone acetate.

Progesterone is a steroid sex hormone found endogenously in both males and females. In females, it modulates the menstrual cycle and maintains pregnancy; in both genders it is known to exert cerebroprotective effects and potentiate neural myelination [1,2]. Progesterone and its synthetic variants are commonly prescribed as drugs for a range of female indications, including hormone replacement therapy (HRT), contraception, endometriosis and certain hormone-responsive cancers [3]. Progesterone, when taken orally for release in the upper gastrointestinal (GI) tract, has very low bioavailability due to poor absorption and extensive first-pass metabolism. There is also considerable variability in the pharmacokinetics (PK) of progesterone between individuals [4]. Medroxyprogesterone acetate (MPA) and levonorgestrel (LNG) are two common synthetic progestogens (Figure 1). Although, by design, MPA and LNG have slightly improved PK profiles compared to progesterone, they still commonly cause GI side effects, show significant inter-individual variability in plasma concentrations, and undergo substantial first-pass metabolism by cytochrome P450 (CYP450) enzymes [5,6,7].

Though CYP450 metabolism is commonly described as occurring in the liver, these enzymes are also highly expressed in mature enterocytes of the gut wall [8,9]. In the case of the CYP3A subfamily, which is known to metabolize the progestogens, enzyme expression in the GI tract may even exceed that in the liver [10]. This high concentration of enzymes in the upper GI tract could contribute to the poor oral bioavailability of prescribed progestogens. Therefore, orally-administered progesterone, LNG, and MPA may benefit from being targeted to the colon, where concentration of metabolizing enzymes is much lower than in the proximal gut [10]. Drugs can be easily targeted to the colon using coatings that resist formulation disintegration in the upper GI tract [11,12]. In the past, it has been shown that the lipid-lowering drug simvastatin is afforded significantly higher bioavailability when delivered to the colon, due to reduced CYP450 exposure [10].

Despite showing promise for colonic delivery, it is currently not known how progesterone, LNG, and MPA fare in colonic conditions. The colon houses trillions of bacteria, fungi, viruses, and protozoa, known collectively as the microbiota [13,14,15]. The colon has the highest density of microbiota of the whole GI tract and thus presents different implications for drug bioavailability compared to the small intestines [16,17,18]. For decades, the metabolic capacity of the gut microbiota has been equated to that of the liver [19,20]. Over 170 orally-administered drugs are currently known to be substrates for enzymes produced by gut bacteria [21,22,23]. It is therefore important to investigate drugs’ susceptibility to microbiota metabolism before formulating for colonic delivery [24,25]. This study will examine the extent to which progesterone, LNG, and MPA may be degraded in the colon, using human fecal slurry. Human feces are commonly employed as a proxy for the colonic microbiota, as supply is convenient and non-invasive [26,27,28,29]. Results will ascertain whether targeting these drugs to the colon is a practical option. This work could lead to new formulations of the drugs with improved PK profiles.

## 2. Materials and Methods

### 2.1. Materials

Progesterone, MPA and LNG were purchased from Sigma Aldrich, Dorset, UK. Peptone water and yeast extract were obtained from Oxoid Limited (Basingstoke, UK). Sodium chloride and dipotassium hydrogen phosphate were obtained from Fisher Chemical (Loughborough, UK). Magnesium sulphate heptahydrate and calcium chloride hexahydrate were obtained from VWR (Lutterworth, UK). Sodium bicarbonate was from Sigma Aldrich, while hemin, l-cysteine HCl, vitamin K and Resazurin were obtained from Sigma Life Sciences (Dorset, UK). Bile salts and tween 80 were from Fluka Analytical and Sigma Aldrich, UK. Methanol was purchased from Fisher scientific and was of HPLC analytical grade. Water was purified using Elga reservoir water purification system. All other chemicals and solvents were of HPLC reagent grade and were used without further purification.

### 2.2. Preparation of Drug Stock Solution

Drug stock solutions were prepared on the day of the experiment. Stock solutions of the three drugs were prepared in DMSO (50%), tween 80 (12.5%) and water (37.5%). The initial stock solution of all three drugs was at a concentration of 1.3 mM. The final concentration of drugs incubated in the simulated colonic fluid (fecal slurry) was 65 µM and the final concentration of tween 80 and DMSO in the slurry was at 1%. All three drugs were shown to be stable for least 24 h in the stock solution.

### 2.3. Preparation of Basal Medium and Phosphate Buffer

Basal medium was prepared to support aerobic and anaerobic bacterial growth in the fecal slurry. This allows for acute prediction of the hormones’ degradation by colonic bacteria over 24 h of incubation. Peptone water and yeast extract (3 g) were weighed into a glass flask containing distilled water (1.3 L) and the solution was autoclaved at 125 °C for 20 min. Separately, calcium chloride hexahydrate (0.01 g), dipotassium hydrogen orthophosphate (0.06 g), magnesium sulphate heptahydrate (0.015 g), and sodium chloride (0.15 g) were dissolved by stirring in distilled water (ca. 150 mL) in a 200 mL volumetric flask. Tween 80 (3 mL), l-cysteine and bile salts (0.75 g) were then added and subsequently dissolved in the same solution. Into this, hemin (0.0075 g in two drops of 1 M sodium hydroxide (NaOH)), 0.025% resazurin in deionized water solution (6 mL), and vitamin K (15 µL) were added under stirring. Upon dissolution, sodium bicarbonate (3 g) and distilled water were added to make the final volume of solution up to 200 mL. Between additions, the flask was stoppered to avoid dissolution of oxygen during stirring. The resultant 200 mL solution was transferred to a laminar flow cabinet and filtered using 0.45 μm Millex GP syringe-driven filter units (Millipore, Carrigtwohill, Ireland). Following filtration, the solution was aseptically combined with the peptone water and yeast extract solution. This final basal medium was stored at room temperature and tightly sealed until use. Any basal medium stored for over 7 days or showing signs of sedimentation was discarded. British Pharmacopoeia specifications were followed to prepare phosphate buffer saline. Potassium dihydrogen orthophosphate (0.014 M), dipotassium hydrogen orthophosphate trihydrate (0.017 M) and sodium chloride (0.256 M) were dissolved in distilled water (2 L) by stirring. The pH of this solution was subsequently adjusted to 6.80 using 0.1 M hydrochloric acid or 1 M NaOH solutions.

### 2.4. Preparation of Fecal Slurry

Fecal samples were provided by two healthy male volunteers in sterile plastic containers. The volunteers were not taking any prescribed medicines, nor had taken any antibiotics in the previous 6 months. This is important as many antibiotic and non-antibiotic drugs are known to alter the gut microbiome [30]. The freshly voided human feces were transferred from their sterile container into an anaerobic workstation (Electrotek 500TG workstation, Electrotek, West Yorkshire, UK) set at a temperature of 37 °C and 70% relative humidity. A total of 20% (*w*/*w*) fecal slurry was prepared by homogenizing the fecal material (60 g) with phosphate buffer saline pH 6.80 (240 g) using an Ultra Turrax (IKA T18 Basic) homogenizer at a speed of 18,000 rpm. Homogenization was judged complete when slurry visually contained no large solid agglomerates. As a secondary measure, the slurry was sieved through 350 µm nylon mesh (Sefar NitexTM, Heiden, Switzerland) to ensure removal of any fibrous material. A 1:1 dilution of the homogenized fecal slurry (300 g) and basal medium (300 g) was then prepared. This final fecal slurry (simulated human colonic fluid) used for the drug stability studies was thus composed of 10% human feces, 40% phosphate buffer saline, and 50% basal medium.

### 2.5. Experimental Procedure

Stability studies of the drugs in simulated human colonic fluid were carried out within an anaerobic workstation set at a temperature of 37 °C and 70% relative humidity. Drug stock solutions at a concentration of 1.3 mM were combined with fecal slurry to achieve a final concentration of 65 µM. The drug-slurry mixture was agitated throughout the experiment on a horizontal shaker at 100 rpm (VXR basic Vibrax^®^, Leicestershire, UK) to mimic colonic conditions. Samples of the drug-slurry mixture were withdrawn at 0, 10, 20, 30, 60, 120, 240 and 360 min and final sample was withdrawn at 24 h. Following withdrawal, samples were added to ice-cold acetonitrile at a ratio of 1:3 to stop any further degradation of drug by inactivating enzymes and to precipitate other proteins that could interfere with analysis. The withdrawn samples were subsequently centrifuged at 10,000 rpm for 10 min. The supernatant was collected and stored at 4 °C until HPLC analysis. Control studies were carried out using the same method, using phosphate buffer saline pH 6.80 in the place of fecal slurry.

### 2.6. HPLC Analysis

The same HPLC method was used to analyze all 3 drugs, in both fecal slurry and control medium. A Hewlett Packard 1100 series HPLC Value System with G1312A binary pump, G1313A Autosampler, and G1315A diode array detector was connected to a Dell Dimension Dim 300 PC for computational analysis using Chemstation for LC 3D systems software (Agilent, Santa Clara, USA). Samples were eluted isocratically in a mobile phase of 80:20 methanol and water. A total of 100 µL of the sample was injected for each run, and passed through a Phase Separations 250 × 4.6 mm licrosphere column (5 µM particle size) at a flow rate of 1.0 mL/min and a temperature of 40 °C. To reduce runover samples were allowed to run for 60 min. The retention time for progesterone, MPA and LNG was 8.6 min, 6.8 min and 7 min, respectively, and was calculated by assessing the time in minutes from sample injection to the maximum point of the elution peak on the HPLC system. The drug concentration within samples was determined using a UV detector wavelength of 242 nm.

### 2.7. Data Analysis

Degradation studies for each drug within the simulated human colonic fluid were repeated thrice, thus, the results presented are the averages (means) and corresponding standard deviations. The percentage of the remaining intact drug within samples at each timepoint was determined by taking the concentration of the drug at the initial time point as 100%. The half-lives (t_1/2_) and rate constants (k) of the drugs were determined by fitting first-order kinetic models by least squares minimization to percentage of the drug in samples vs. incubation time curves (Origin 8.1). The Kolmogorov-Smirnov test was used to assess data distribution for normality, and ANOVA followed by t-tests determined the statistical significance between groups (SPSS version 10.0, Chicago, USA). Significance was justified using a threshold of *p*-value < 0.05.

## 3. Results and Discussion

**Figure 2:**
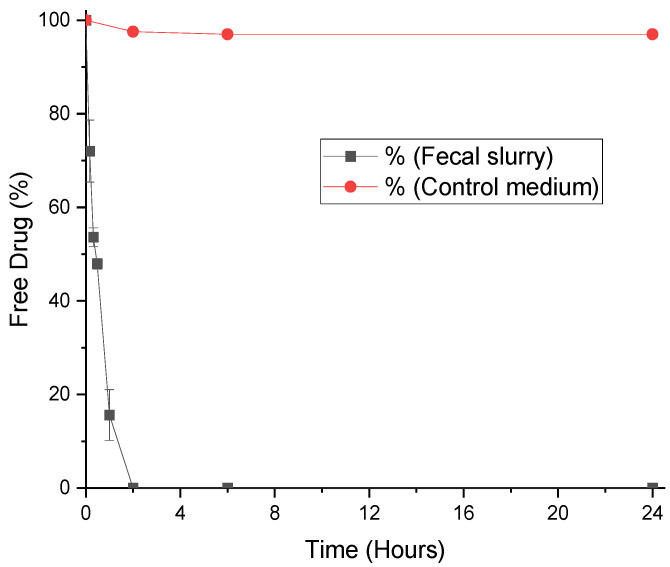
Stability profile of progesterone in human fecal slurry (mean ± S.D.) and control medium.

**Figure 3:**
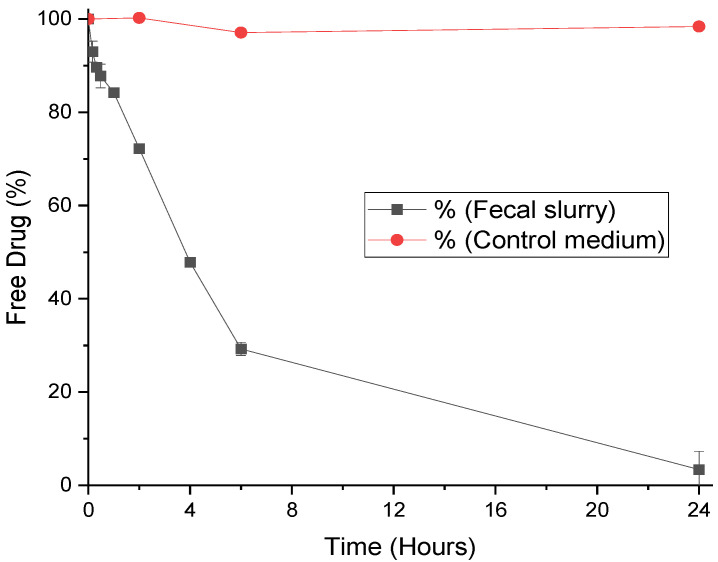
Stability profile of levonorgestrel in human fecal slurry (mean ± S.D.) and control medium.

**Figure 4:**
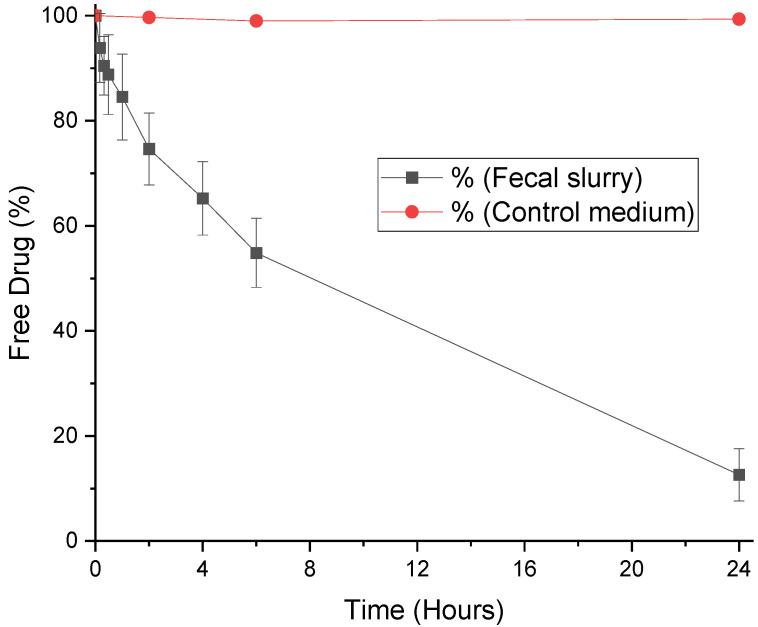
Stability profile of medroxyprogesterone acetate in human fecal slurry (mean ± S.D.) and control medium.

The stabilities of progesterone, MPA, and LNG were assessed in fecal slurry for 24 h. The degradation of each hormone in fecal slurry can be seen in Figure 2, Figure 3 and Figure 4, and half-lives are presented with rate constants in Table 1. The drugs remained 99% detectable after 24 h in control media, confirming that decreases in the hormone concentration within the simulated colonic fluid were due to microbiota. As steroid hormones, the progestogens have been found to behave in a comparable manner to three corticosteroids. Prednisolone, budesonide, and beclomethasone dipropionate have been proven in the past to be extensively metabolized in simulated colonic fluid as well [26]. Moreover, there is evidence that the steroid hormones cortisol and estrogens are degraded by gut microbiota [31,32]. Interestingly, whilst the gut microbiome clearly plays a role in hormone metabolism, a number of studies have found a role for hormones in shaping gut microbiome composition, elucidating a bidirectional relationship [31,33]. From a clinical viewpoint, it is anticipated that microbiome metabolism could affect systemic drug exposure and thus lead to inter-individual variations in PK [34,35,36]. Progesterone, MPA, and LNG are known to show such inter-patient variations, a challenge that may make the selection of dosage regimens difficult. Understanding the kinetics of microbiome metabolism is therefore of key importance.

### 3.1. Degradation of Progesterone by Fecal Microbiota

Progesterone was degraded most rapidly amongst the three hormones, with undetectable levels in fecal slurry after 2 h (Figure 2). The reaction rate was fast: after 60 min only 12% of progesterone remained in the medium. The half-life in these conditions was calculated as just 28 min (Table 1). Progesterone can be metabolized to more than 30 compounds in vivo [37]. The most prominent pathway is the conversion to 5α- and 5β-pregnanolone, compounds that can bind to cerebral GABA_A_ receptors and cause considerable sedative side effects [38]. Other important metabolites include 20-dihydroprogesterone (with weak progestogenic activity), 11-deoxycorticosterone (a potent mineralocorticoid), and 17α-hydroxyprogesterone (inactive) [37]. Gut bacteria are known to predominately perform hydrolytic and reductive reactions, therefore, it is reasonable to predict that 5α- and 5β-pregnanolone are produced by gut bacteria through reduction [39,40]. Progesterone’s other known metabolites are more likely produced through oxidation by CYP450 enzymes in the gut wall and liver [41]. Based on this work, progesterone is not an ideal drug for colonic delivery, as bioavailability will likely be impacted by rapid and extensive metabolism by microbiota.

### 3.2. Degradation of LevonorGestrel by Fecal Microbiota

LNG had a half-life of 240 min in the fecal medium (Table 1). Degradation of LNG by microbiota was more gradual than progesterone, with a reaction rate nearly ten-fold smaller (Table 1). After 6 h in the presence of fecal microbiota, over 29% of LNG was still intact (Figure 3). This shows that the synthetic structure of LNG protects it from degradation, due to decreased affinity with microbiota enzymes. The structural necessities for binding affinity to progesterone receptors and the resultant progestogenic activity are the presence of a 3-keto group and a double bond between C4 and C5 on the same aromatic ring (Figure 1). LNG is the biologically active enantiomer of the racemic synthetic hormone norgestrel, and differs from progesterone by its ethinyl group at C17 and lack of methylation on the ketone ring [42,43]. These structural differences afford it comparably higher androgenic activity compared to progesterone, due to molecular similarities with testosterone [37].

Known metabolites of LNG include several tetrahydro- and hydroxy- derivatives, in addition to sulfate and glucuronide conjugations produced during phase II hepatic metabolism [42,44]. Whilst the hydroxylations of LNG are probably CYP450-mediated, it is possible that the ring-reduced tetrahydro-LNG compounds are formed by gut microbiota. Therefore, LNG delivered to the colon will likely be metabolized differently, and potentially more slowly, than in the upper GI tract in the presence of CYP450 enzymes. This study shows the stability of LNG in fecal microbiota, supporting its colonic targeting for improvement of bioavailability after oral administration.

### 3.3. Degradation of Medroxyprogesterone Acetate by Fecal Microbiota

Of the three progestogens studied, MPA by far displayed the highest stability in fecal slurry. With a half-life of 644 min, over 12% of MPA was present even after 24 h (Figure 4, Table 1). MPA differs structurally from progesterone by acetylation at C17 and the addition of a methyl group at C6. These differences clearly decrease the suitability of the drug as a substrate for microbiota enzymes, as represented by a slower rate of degradation (Table 1). Mass spectrometry has elucidated 5β-dihydro MPA and tetrahydro MPA as key metabolites formed in the presence of gut bacteria [45]. These are reduction reactions that attack the polar ketone bond on aromatic ring A of the hormone. In comparison, CYP3A4 enzymes are thought to oxidize the drug, forming 6β-, 2β-, and 1β-hydroxy MPA, whereby hydroxyl groups are added to rings A or B [46,47]. This suggests that the pharmacokinetics of MPA will be different depending on where it is delivered in the GI tract. MPA delivered to the small intestines is likely to undergo degradation by CYP450 enzymes in the gut wall; indeed, a recent clinical study showed orally administered MPA has limited activity due to its poor bioavailability, despite dose escalation [48]. In juxtaposition, the colon has significantly lower levels of CYP450 enzymes, and higher concentrations of microbiota [16]. MPA is a known substrate for CYP450 enzymes, therefore, its stability in human fecal slurry makes it a promising candidate for colonic drug targeting.

A limitation of this study is the lack of variety of microbiome samples used to measure drug stability. Gut microbiota composition is known to be influenced by age, gender, geographical location, medication use, diet, health, and many other factors [49]. These inter-individual differences can lead to variability in microbiome-mediated metabolism of drugs [21]. Whilst there is no current blueprint for a “healthy” microbiome, it is thought that a healthy ‘”functional core” is more likely, whereby similar functions are performed between healthy individuals’ microbiomes, though not necessarily performed by the same species and strains of microbiota [49]. This study therefore aims to provide a model for how the progestogens are degraded by healthy “functional core” microbiota, to assess the feasibility of formulation for colonic delivery. In future work, the degradation of colon-targeted progestogens can be assessed in a larger sample size of microbiomes.

## 4. Conclusions

This study measured the degradation kinetics of progesterone, LNG, and MPA during incubation in human fecal slurry, to assess degradation by colonic microbiota. The half-lives of progesterone, MPA, and LNG in fecal slurry were 28, 644, and 240 min, respectively. The rate of degradation of progesterone was the fastest, followed by LNG, and then MPA. The expected biotransformation pathways of the three hormones were explored, with gut bacteria predicted to be responsible for hydrolytic and reductive reactions. This work promotes formulation of LNG and MPA, but not progesterone, for delivery to the colon. By circumventing the upper GI tract, oral formulations of LNG and MPA may avoid metabolism by gut CYP3A enzymes, improving their current low bioavailabilities and variable PK profiles. Colonic delivery of these drugs could therefore provide advantages over current delivery methods, thus improving patient outcomes.

## Figures and Tables

**Table 1:** Rate constant and half-lives (t_1/2_) of progesterone, medroxyprogesterone acetate (MPA) and levonorgestrel (LNG).

Hormone	Rate Constant (min^−1^)	t ½ (Minutes)
**Progesterone**	0.0316 ± 0.0054	28.25 ± 0.42
**MPA**	0.0029 ± 0.0016	644.18 ± 34.95
**LNG**	0.0029 ± 0.0002	240.38 ± 3.44
